# Prevalence of orofacial clefts in Latin America and the Caribbean: trends between 2000 and 2020

**DOI:** 10.17843/rpmesp.2024.412.13558

**Published:** 2024-05-27

**Authors:** Juan Sebastián Zuluaga-Morales, Brenda Yuliana Herrera-Serna, Olga Patricia López-Soto, Gloria María Sandoval-Llanos, Juliana Martínez-Nieto

**Affiliations:** 1 Dentistry Program, Universidad Autónoma de Manizales. Manizales, Colombia. Universidad Autonoma de Manizales Dentistry Program Universidad Autónoma de Manizales Manizales Colombia

To the Editor. Orofacial clefts (OFC) are the most common congenital defect affecting the head and neck. Currently, there are few studies of prevalence and trends of OFC in Latin America, and even fewer studies comparing the burden of disease among representative countries of the region. The identification of temporal variation in the prevalence of OFC may reflect changes in environmental risk factors and, in turn, provide a relevant basis for future prevention and control strategies.

We conducted research following 15 of the 18 Guidelines for Accurate and Transparent Health Estimates Reporting (GATHER) ^1^^)^ with the aim of determining the trends of OFC prevalence rates in Latin America and the Caribbean between 2000 and 2020; the guidelines that were not reported account for estimates made from the original data source. This study was endorsed by the Ethics, Bioethics and Scientific Integrity Committee of the Universidad Autónoma de Manizales as stated in the document No. 2021-126 of 1-12-2021.

Data from the Global Burden of Disease (GBD) study was used, including OFC prevalence rates during the years 2000 to 2019 from 20 countries in Latin America and the Caribbean for men and women, and age-standardized rates (direct adjustment method using the population established by WHO as reference). All data can be obtained from the database (Global Health Exchange - GHDx) on the website http://ghdx.healthdata.org/data-type/disease-registry. Data and trend projections were made for the year 2020. A first-order autoregressive regression analysis was performed to define trends in the prevalence of OFCs between 2000 and 2020. Subsequently, the Joinpoint regression analysis program, version 4.7.0.0, (National Cancer Institute, USA) was used with an overall significance level of p<0.05 to calculate the average annual percentage change (AAPC) and the uncertainty interval.

Table 1 shows that the countries with the highest age-standardized prevalence rates of OFC for both sexes and for the years 2000 and 2020 in Latin America and the Caribbean were Peru, Nicaragua and the Dominican Republic. Most countries showed increasing trends ranging from 0.1 to 1.8 AAPC; except Chile, Mexico and Ecuador, which showed decreasing trends for both sexes.

**Table 1:** Age-standardized prevalence rates and average annual percentage change in prevalence of orofacial clefts by sex and country between 2000 and 2020 in Latin America and the Caribbean.

	Age-standardized prevalence rates of orofacial clefts				AAPC men (II)	Trend	AAPC women (II)	Trend
	Men		Women					
Countries	2000	2020	2000	2020				
Argentina	26.17	27.84	25.00	24.93	0.3* (0.2 to 0.4)	Increasing	0.1 (-0.1 to 0.2)	Stationary
Bolivia	32.46	33.16	26.91	30.73	0.3 (0.2 to 0.3)	Increasing	0.6* (0.6 to 0.7)	Increasing
Brazil	22.50	22.82	19.49	19.14	0.1 (0.0 to 0.3)	Increasing	-0.1* (-0.2 to 0.0)	Stationary
Chile	20.80	19.61	20.95	19.48	-0.4* (-0.5 to -0.2)	Decreasing	-0.3* (-0.3 to -0.2)	Decreasing
Colombia	27.75	32.76	31.40	36.24	0.9* (0.8 to 0.9)	Increasing	0.8* (0.8 to 0.9)	Increasing
Costa Rica	20.47	20.13	20.39	20.98	-0.2* (-0.3 to -0.1)	Decreasing	0.2 (0.0 to 0.4)	Increasing
Cuba	53.62	60.10	22.67	32.88	0.7* (0.6 to 0.8)	Increasing	1.8* (1.7 to 1.8)	Increasing
Dominican Republic	56.50	64.30	32.64	43.13	0.6* (0.6 to 0.7)	Increasing	1.4* (1.3 to 1.5)	Increasing
Ecuador	18.78	17.40	17.46	17.73	-0.4* (-0.6 to -0.3)	Decreasing	-0.1 (-0.3 to -0.1)	Decreasing
El Salvador	29.08	33.90	30.99	32.13	0.8* (0.7 to 0.8)	Increasing	0.3* (0.2 to 0.4)	Increasing
Guatemala	33.75	36.25	32.01	32.61	0.4* (0.3 to 0.4)	Increasing	0.1* (0.1 to 0.2)	Increasing
Haiti	53.75	60.86	30.12	41.25	0.7* (0.6 to 0.7)	Increasing	1.7* (1.6 to 1.7)	Increasing
Honduras	30.71	31.58	30.47	32.45	0.4* (0.2 to 0.6)	Increasing	0.4* (0.4 to 0.5)	Increasing
Mexico	44.89	36.05	33.57	31.22	-1.0* (-1.5 to -0.5)	Decreasing	-0.4 (-1.0 to -0.2)	Decreasing
Nicaragua	49.61	56.16	51.02	54.65	0.7* (0.6 to 0.8)	Increasing	0.4* (0.2 to 0.6)	Increasing
Panama	30.38	34.69	30.43	33.54	0.6* (0.6 to 0.7)	Increasing	0.5* (0.4 to 0.7)	Increasing
Paraguay	17.00	18.00	13.85	15.25	0.2* (0.1 to 0.2)	Increasing	0.4* (0.4 to 0.4)	Increasing
Peru	61.21	67.72	47.75	55.95	0.5* (0.5 to 0.5)	Increasing	0.8* (0.7 to 0.8)	Increasing
Uruguay	22.21	22.54	21.26	21.80	0.1 (-0.1 to 0.2)	Stationary	0.2* (0 to 0.3)	Increasing
Venezuela	29.89	33.69	30.34	31.55	0.7* (0.6 to 0.7)	Increasing	0.3* (0.1 to 0.4)	Increasing

AAPC: average annual percentage change; II: uncertainty interval; *statistically significant (p<0.05). The statistical test shows the mean of the forecast f for the regression.

This could be due to prenatal diagnostic techniques, including fetal ultrasound, fetal echocardiography and karyotyping after amniocentesis and chorionic villus sampling that allow diagnosis of severe structural damage before the beginning of the second trimester (usually before 22 weeks of gestation) ^2^. In addition, most mothers carrying a fetus affected by severe malformation would decide to choose gestational termination ^2^. Therefore, these children with OFC, according to current medical practices, would not be born and thus, the prevalence figures would decrease.

Countries with comparatively low gross domestic product (GDP) in Latin America, such as Haiti, Nicaragua and Bolivia, had a high age-standardized prevalence rate of OFC. There is much controversy about the likely correlation between socioeconomic factors and OFC, due to the methodological diversity found in different study designs. For example, the study by Vrijheid *et al*. found no evidence that socioeconomic factors could interfere with or increase cases of OFC ^3^. For their part, Womersley & Stone ^4^ observed in 1987 that teratogenic factors were more prevalent in areas of lower socioeconomic levels, where unhealthy environmental conditions increased susceptibility to a specific teratogen, possibly causing facial clefts.

Ethnicity has also been related to OFC. Thus, Amerindian ethnicity (prevalent in Bolivia, Patagonia, Ecuador, and Argentina), and altitude above sea level (higher in Bolivia and Ecuador) are associated with clusters of high OFC prevalence at birth ^5^. It is important to consider that cases could be underestimated in regions with the lowest prevalence of OFC. Another relevant aspect is the misdiagnosis of orofacial clefts, mainly those associated with cleft palate syndromes and cases ^6^.

Given that we used data from the GBD as a secondary source, one of the limitations of this study is that there was no control over the quality of the information. However, the effort made by the GBD for the robust and exhaustive estimation of the data should be recognized. In addition; due to their design, ecological studies are not able to associate exposure and disease at the individual level, since the data collected represent the mean of exposure levels rather than individual values.

In conclusion, the distribution of OFC in Latin American and Caribbean countries is heterogeneous and there is no geographic pattern. Countries such as Peru, Haiti and the Dominican Republic have shown greater affectation, and coincide with their increasing trends; while Chile and Ecuador showed the main decreasing trends.
