# Supplementary material for: Prevalence of orofacial clefts in Latin America and the Caribbean: trends between 2000 and 2020
Source: Rev Peru Med Exp Salud Publica. 2024 May 27;41(2):220–2. doi: 10.17843/rpmesp.2024.412.13558 (PMC11300698; doi:10.17843/rpmesp.2024.412.13558)
Supplement: Translation

# Translation: Prevalencia de fisuras orofaciales en Latinoamérica y el Caribe: tendencias entre 2000 y 2020

**Authors:** Juan Sebastián Zuluaga-Morales, Brenda Yuliana Herrera-Serna, Olga Patricia López-Soto, Gloria María Sandoval-Llanos, Juliana Martínez-Nieto

**Affiliations:** 1 Programa de Odontología, Universidad Autónoma de Manizales. Manizales, Colombia.

Sr. Editor. Las fisuras orofaciales (FOF) son el defecto congénito más común que afecta la cabeza y el cuello. Hasta ahora, son pocos los estudios de prevalencia y tendencias de FOF en Latinoamérica, y más escasos aún, aquellos que comparen la carga de enfermedad entre países representativos de dicha región. La identificación de la variación temporal en las prevalencias de las FOF puede reflejar modificaciones en los factores de riesgo ambientales, y a su vez, brindar bases relevantes para futuras estrategias de prevención y control.

Con el objetivo de determinar las tendencias de la prevalencia de FOF en Latinoamérica y el Caribe entre 2000 y 2020, se realizó una investigación siguiendo 15 de las 18 Directrices para la Elaboración de Informes de Estimaciones de Salud Precisas y Transparentes (GATHER, en inglés) ^1^, de las cuales aquellas que no se reportan dan cuenta de las estimaciones realizadas desde la fuente original de los datos. Este estudio contó con el aval del Comité de Ética, Bioética e Integridad Científica de la Universidad Autónoma de Manizales como consta en el acta Nro. 2021-126 del 1-12-2021.

Se utilizaron datos del estudio de Carga Global de la Enfermedad (GBD, en inglés), se incluyó la prevalencia durante los años 2000 a 2019 para FOF en 20 países de Latinoamérica y el Caribe para hombres y mujeres, y las tasas estandarizadas por edad (método de ajuste directo usando como población de referencia, la establecida por la OMS). Todos los datos se pueden obtener de la base de datos (Global Health Exchange - GHDx) en el sitio web http://ghdx.healthdata.org/data-type/disease-registry. Se realizaron proyecciones de los datos y las tendencias para el año 2020. Para definir las tendencias en la prevalencia de las FOF entre 2000 y 2020 se realizó un análisis de regresión autorregresivo de primer orden. Posteriormente, para calcular el cambio porcentual anual promedio (AAPC, en inglés) y el intervalo de incertidumbre, se utilizó el programa de análisis de regresión Joinpoint, versión 4.7.0.0, (Instituto Nacional del Cáncer, EE. UU.) con un nivel de significación global de p<0,05.

La Tabla 1 muestra que los países con mayores tasas estandarizadas por edad de prevalencia de FOF para ambos sexos y para los años 2000 y 2020 en Latinoamérica y el Caribe fueron Perú, Nicaragua y República Dominicana. La mayoría de los países mostraron tendencias crecientes que oscilaron entre AAPC 0,1 y 1,8; excepto Chile, México y Ecuador que evidenciaron tendencias decrecientes para ambos sexos.

**Tabla 1:** Tasas estandarizadas por edad de prevalencia y cambio porcentual anual promedio de la prevalencia de fisuras orofaciales por sexo y país entre 2000 y 2020 en Latinoamérica y el Caribe.

	Tasas estandarizadas por edad de prevalencia de fisuras orofaciales				AAPC hombres (II)	Tendencia	AAPC mujeres (II)	Tendencia
	Hombres		Mujeres					
Países	2000	2020	2000	2020				
Argentina	26,17	27,84	25,00	24,93	0,3* (0,2 a 0,4)	Creciente	0,1 (-0,1 a 0,2)	Estacionaria
Bolivia	32,46	33,16	26,91	30,73	0,3 (0,2 a 0,3)	Creciente	0,6* (0,6 a 0,7)	Creciente
Brasil	22,50	22,82	19,49	19,14	0,1 (0,0 a 0,3)	Creciente	-0,1* (-0,2 a 0,0)	Estacionaria
Chile	20,80	19,61	20,95	19,48	-0,4* (-0,5 a -0,2)	Decreciente	-0,3* (-0,3 a -0,2)	Decreciente
Colombia	27,75	32,76	31,40	36,24	0,9* (0,8 a 0,9)	Creciente	0,8* (0,8 a 0,9)	Creciente
Costa Rica	20,47	20,13	20,39	20,98	-0,2* (-0,3 a -0,1)	Decreciente	0,2 (0,0 a 0,4)	Creciente
Cuba	53,62	60,10	22,67	32,88	0,7* (0,6 a 0,8)	Creciente	1,8* (1,7 a 1,8)	Creciente
República Dominicana	56,50	64,30	32,64	43,13	0,6* (0,6 a 0,7)	Creciente	1,4* (1,3 a 1,5)	Creciente
Ecuador	18,78	17,40	17,46	17,73	-0,4* (-0,6 a -0,3)	Decreciente	-0,1 (-0,3 a -0,1)	Decreciente
El Salvador	29,08	33,90	30,99	32,13	0,8* (0,7 a 0,8)	Creciente	0,3* (0,2 a 0,4)	Creciente
Guatemala	33,75	36,25	32,01	32,61	0,4* (0,3 a 0.4)	Creciente	0,1* (0,1 a 0,2)	Creciente
Haití	53,75	60,86	30,12	41,25	0,7* (0,6 a 0,7)	Creciente	1,7* (1,6 a 1,7)	Creciente
Honduras	30,71	31,58	30,47	32,45	0,4* (0,2 a 0,6)	Creciente	0,4* (0,4 a 0,5)	Creciente
México	44,89	36,05	33,57	31,22	-1,0* (-1,5 a -0,5)	Decreciente	-0,4 (-1,0 a -0,2)	Decreciente
Nicaragua	49,61	56,16	51,02	54,65	0,7* (0,6 a 0,8)	Creciente	0,4* (0,2 a 0,6)	Creciente
Panamá	30,38	34,69	30,43	33,54	0,6* (0,6 a 0,7)	Creciente	0,5* (0,4 a 0,7)	Creciente
Paraguay	17,00	18,00	13,85	15,25	0,2* (0,1 a 0,2)	Creciente	0,4* (0,4 a 0,4)	Creciente
Perú	61,21	67,72	47,75	55,95	0,5* (0,5 a 0,5)	Creciente	0,8* (0,7 a 0,8)	Creciente
Uruguay	22,21	22,54	21,26	21,80	0,1 (-0,1 a 0,2)	Estacionaria	0,2* (0 a 0,3)	Creciente
Venezuela	29,89	33,69	30,34	31,55	0,7* (0,6 a 0,7)	Creciente	0,3* (0,1 a 0,4)	Creciente

AAPC: cambio porcentual anual promedio (en inglés); II: intervalo de incertidumbre; *estadísticamente significativo (p<0,05). La prueba estadística muestra la media del pronóstico f para la regresión.

Esto podría deberse a las técnicas de diagnóstico prenatal, incluyendo ecografía fetal, ecocardiografía fetal y cariotipo después de amniocentesis y vellosidades coriónicas que permiten diagnosticar daños estructurales severos antes de iniciar el segundo trimestre (generalmente antes de 22 semanas de gestación) ^2^. Además, la mayoría de las madres que llevan un feto afectado por una malformación grave, decidirían elegir la terminación de la gestación ^2^. Por lo tanto, estos niños con FOF, según las prácticas médicas actuales no alcanzarían a nacer y las cifras de prevalencia disminuyen.

Los países con un producto interno bruto (PIB) bajo en Latinoamérica, comparativamente, como Haití, Nicaragua y Bolivia presentaron una tasa de prevalencia de FOF estandarizadas por edad altas. Existe una gran controversia sobre la probable correlación entre los factores socioeconómicos y las FOF, debido a la diversidad metodológica encontrada en los diferentes diseños de estudio. Por ejemplo, el estudio de Vrijheid *et al.,* no encontró evidencia de que los factores socioeconómicos pudieran interferir o aumentar los casos de FOF ^3^. Por su parte, Womersley & Stone ^4^ desde 1987 observaron que los factores teratogénicos eran más prevalentes en zonas de mayor carencia socioeconómica, donde las condiciones ambientales insalubres aumentaban la susceptibilidad a un teratógeno específico, posiblemente causando hendiduras faciales.

El origen étnico también ha sido relacionado con las FOF.​ Es así como el origen étnico amerindio (prevalente en Bolivia, Patagonia, Ecuador, y Argentina), y la altitud sobre el nivel del mar (mayor en Bolivia y Ecuador) se asocian a conglomerados de alta prevalencia de FOF en el nacimiento ^5^. Es importante considerar que, en aquellas regiones que muestran la prevalencia más baja de FOF, los casos podrían estar subestimados. Otro aspecto relevante es el diagnóstico erróneo de hendiduras orofaciales, principalmente las asociadas a síndromes y casos de paladar fisurado ^6^.

En vista de que este estudio utilizó la GBD como fuente secundaria, una de las limitaciones identificadas, es que no se tiene control sobre la calidad de la información. Sin embargo, se debe reconocer el esfuerzo que realiza el GBD para la estimación robusta y exhaustiva de los datos. Además; en los estudios ecológicos, no es posible asociar la exposición y la enfermedad a nivel individual, dado que los datos recogidos representan la media de niveles de exposición en lugar de valores individuales.

En conclusión, la presentación de FOF en países de Latinoamérica y el Caribe es heterogénea y no se observa un patrón de distribución geográfica. Países como Perú, Haití y República Dominicana, han mostrado mayor afectación, y coinciden con sus tendencias crecientes; mientras que Chile y Ecuador han mostrado las principales tendencias decrecientes.
